# Noninvasive, Multimodal Inflammatory Biomarker Discovery for Systemic Inflammation (NOVA Study): Protocol for a Cross-Sectional Study

**DOI:** 10.2196/62877

**Published:** 2024-11-05

**Authors:** Jinjoo Shim, Sinziana Muraru, Rucsandra Dobrota, Elgar Fleisch, Oliver Distler, Filipe Barata

**Affiliations:** 1 Centre for Digital Health Interventions ETH Zurich Zurich Switzerland; 2 Department of Rheumatology University Hospital Zurich University of Zurich Zurich Switzerland; 3 Centre for Digital Health Interventions University of St. Gallen St. Gallen Switzerland

**Keywords:** systemic inflammation, chronic inflammation, inflammatory biomarkers, biofluids, serum, urine, sweat, saliva, exhaled breath, stool, C-reactive protein, interleukin, IL-1β, IL-6, IL-8, IL-10, tumor necrosis factor, TNF-α, fractional exhaled nitric oxide, calprotectin, core body temperature, noninvasive biomarker, multimodal biomarker, remote monitoring, surrogate biomarker, rheumatology, chronic inflammatory disease

## Abstract

**Background:**

Prolonged systemic inflammation is recognized as a major contributor to the development of various chronic inflammatory diseases. Daily measurements of inflammatory biomarkers can significantly improve disease monitoring of systemic inflammation, thus contributing to reducing the burden on patients and the health care system. There exists, however, no scalable, cost-efficient, and noninvasive biomarker for remote assessment of systemic inflammation. To this end, we propose a novel, multimodal, and noninvasive approach for measuring inflammatory biomarkers.

**Objective:**

This study aimed to evaluate the relationship between the levels of inflammatory biomarkers in serum (gold standard) and those measured noninvasively in urine, sweat, saliva, exhaled breath, stool, and core body temperature in patients with systemic inflammation.

**Methods:**

This study is a single-center, cross-sectional study and includes a total of 20 participants (10 patients with systemic inflammation and 10 control patients). Eligible participants provide serum, urine, sweat, saliva, exhaled breath, and stool samples for biomarker analyses. Core body temperature is measured using a sensor. The primary end point is the level of C-reactive protein (CRP). The secondary end points are interleukin (IL)–1β, IL-6, IL-8, IL-10, and tumor necrosis factor-α levels. The tertiary end points are fractional exhaled nitric oxide, calprotectin, and core body temperature. Samples will be collected in 2 batches, enabling preliminary analysis of the first batch (patients 1-5 from each group). The full analysis will include both batches. CRP and cytokine levels will be measured using enzyme-linked immunosorbent assay and electrochemiluminescence immunoassay. For statistical analysis, the Shapiro-Wilk test will be used to evaluate the normality of the distribution in each variable. We will perform the 2-tailed *t* test or Wilcoxon rank sum test to compare the levels of inflammatory biomarkers between patients with systemic inflammations and control patients. Pearson and Spearman correlation coefficients will assess the relationship between inflammatory biomarkers from noninvasive methods and serum biomarkers. Using all-subset regression analysis, we will determine the combination of noninvasive methods yielding the highest predictive accuracy for serum CRP levels. Participants’ preferences for sampling methods will be assessed through a questionnaire.

**Results:**

The study received ethics approval from the independent research ethics committee of Canton Zurich on October 28, 2022. A total of 20 participants participated in the study measurements. Data collection started on February 22, 2023, and was completed on September 22, 2023. Participants were on average 52.8 (SD 14.4; range 24-82) years of age, and 70% (14/20) of them were women. The analysis results reporting findings are expected to be published in 2025.

**Conclusions:**

This study aims to evaluate the feasibility of noninvasive, multimodal assessment of inflammatory biomarkers in patients with systemic inflammation. Promising results could lead to the creation of noninvasive and potentially digital biomarkers for systemic inflammation, enabling continuous monitoring and early diagnosis of inflammatory activity in a remote setting.

**International Registered Report Identifier (IRRID):**

DERR1-10.2196/62877

## Introduction

Chronic systemic inflammation is a persistent upregulation of inflammatory activity as a result of prolonged exposure to a pathogen or autoimmune dysfunction. Prolonged systemic inflammation is associated with the disease onset and progression of various chronic inflammatory diseases such as rheumatoid arthritis, spondyloarthritis, or systemic lupus erythematosus [1].

Current standardized practice to assess the presence of systemic inflammation and inflammatory disease activity is based on serum biomarkers obtained from a blood test [2]. Increased levels of proinflammatory markers such as the C-reactive protein (CRP) and interleukin (IL)–6, IL-8, and tumor necrosis factor (TNF)–α are closely associated with active inflammation and disease progression in cardiovascular and autoimmune diseases [3]. The serum CRP is found to be the most precise and objective measurement and is regularly assessed at routine hospital visits to confirm the clinical diagnosis, classification of disease activity, and treatment decisions in chronic inflammatory diseases. In the case of rheumatoid arthritis, for instance, clinicians determine the current state of disease activity and treatment response based on aggregated information of the inflammatory biomarker levels from a blood test (eg, CRP), physical assessment (eg, joint counts), and composite score (eg, Disease Activity Score-28) obtained during a routine visit [4,5]. While blood tests provide valuable information to diagnose, screen, and monitor various inflammatory biomarkers and clinical conditions, this procedure has three distinct disadvantages: (1) it is invasive and burdensome to patients, (2) it requires trained personnel and is not feasible for self-administration, and (3) real-time monitoring or at-home measurements are not available. In addition, there are a limited number of studies and insufficient evidence to support the validity of detecting CRP or cytokines noninvasively.

Existing studies assess only specific disease populations, biofluids, or biomarker types, limiting the generalizability of findings to a wide patient population of systemic inflammation. Urinary CRP, for example, is mostly researched in patients with urinary tract infections, other urologic conditions, or rejection of transplanted kidneys [6,7]. Likewise, fractional exhaled nitric oxide (FeNO), a product of metabolic and inflammatory processes in the human body, is largely investigated in cancers and respiratory diseases such as asthma, but rarely in other systemic inflammatory conditions [8,9]. Fecal calprotectin is a well-established biomarker for inflammatory bowel disease, yet its clinical relevance to other inflammatory diseases remains largely unexplored [10]. Recent studies have shown encouraging results including significant associations with serum calprotectin in patients with spondyloarthritis compared with matched controls and increased serum calprotectin levels correlated with disease activity in both rheumatoid arthritis and axial spondyloarthritis [11,12]. Furthermore, chronic inflammation contributes to the disruption of homeostatic control mechanisms, such as core body temperature, and results in increased vulnerability to homeostatic dysregulation and diseases [13]. Yet, the underlying mechanisms responsible for this connection are not yet fully understood.

Noninvasive assessment of inflammatory biomarkers could overcome the limitations of the invasive blood draw and improve the lives of patients with chronic inflammatory diseases by enabling continuous disease monitoring. This study demonstrates the potential to use noninvasive methods for developing accessible and less invasive diagnostic tools. These advancements would alleviate multiple burdens from blood tests, contribute to patients’ self-management, and add significant value to clinicians and caregivers by enabling meaningful treatment and effective prevention of future events, a step toward population-wide measurement and personalized prevention.

To date, however, there exists no scalable solution that allows patients to gauge one’s inflammatory state remotely. As a result, many patients might postpone contacting their physicians until late during a disease progression or even until the next routine visit to evaluate inflammatory symptoms and the CRP level. This delay may not only impact negatively on patients’ quality of life but also cause unanticipated consequences or harm in some cases. An option of remotely monitoring symptoms and inflammatory activity could empower patients toward self-management, increase comprehension of the disease, and thus, ensure engagement in treatment adherence and timely intervention.

To address this research gap, we propose a proof-of-concept feasibility study (NOVA study) to evaluate the relationship between the levels of serum inflammatory biomarkers and those measured noninvasively in urine, sweat, saliva, exhaled breath, core body temperature, and stool in patients with systemic inflammation. We hypothesize that the levels of inflammatory biomarkers measured noninvasively in various biofluids can be used to detect and monitor disease activity and are associated with inflammatory biomarkers measured in serum through a blood test. Once this link is established, it would pave the way to the development of digital biomarkers for real-time disease activity prediction and monitoring of the effectiveness of treatments through scalable, cost-effective, and patient-oriented digital solutions. The proposed study aims at targeting the following three main research questions: (1) to what extent is systemic inflammation measured by serum CRP associated with the CRP measured non-invasively in urine, sweat, and saliva? (2) to what extent is systemic inflammation measured by serum cytokines (ie, IL-1β, IL-6, IL-8, IL-10, and TNF-α) associated with cytokines measured noninvasively in urine and sweat? and (3) to what extent is systemic inflammation measured by serum CRP and cytokines associated with noninvasive measures of FeNO from exhaled breath, calprotectin from stool, and core body temperature?

## Methods

### Study Design and Participants

The NOVA study is a 2-group, cross-sectional, single-center observational study aiming to recruit 20 individuals among the hospitalized patients from the rheumatology ward of University Hospital Zurich. The systemic inflammation group and the control group will include 10 participants each. The inclusion criteria are adult persons (aged 18 years or older) who have provision of written informed consent. Patients with systemic inflammation are defined as patients with the inflammatory syndrome (ie, CRP>5 mg/L). The control patients are patients who do not have an inflammatory syndrome (ie, CRP≤5 mg/L). Exclusion criteria are patients with active psychiatric or mental disorders (ie, clinically relevant depression, dementia, and Alzheimer disease), alcohol abuse, or other substance abuse; patients needing immediate treatment with glucocorticoids or other drugs for the suppression of the inflammation until study procedures can be performed; those with language barriers or incapable of understanding and signing an informed consent form; and those with other factors that impair daily functions and make adherence to the study protocol impossible.

### Procedures

#### Recruitment

**Figure 1:**
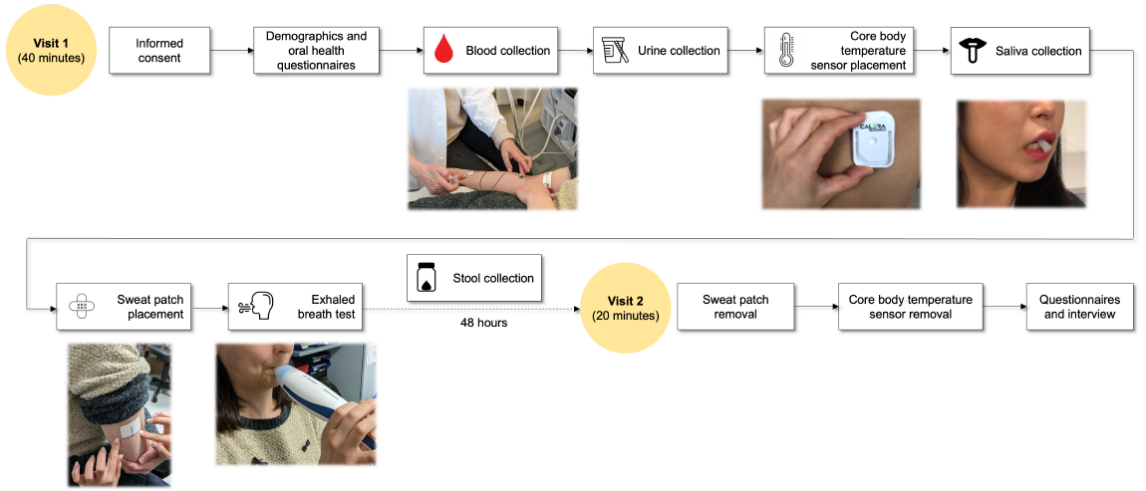
A schematic of NOVA study procedures.

Figure 1 shows the study procedures of the NOVA study. A clinician will conduct a prescreening to identify planned and emergency admissions and check if they have increased CRP levels. In case a patient is deemed eligible for the NOVA study, the clinician or study nurse will contact the patient, hand out a study flyer and a patient information sheet, and ask if they are willing to participate in the study following good clinical practice guidelines. Participants will have 24 hours or overnight to consider study participation before providing informed consent.

#### Visit 1 (Study Measurements)

##### Study Measurements

The study measurements will either take place in the patient’s room or in the clinical examination room available on the ward and are estimated to take approximately 40 minutes. Participants will complete the following steps under the guidance of the study nurse and a researcher from the Centre for Digital Health Interventions (CDHI).

##### Informed Consent

If the eligible patient agrees to participate in the study, a study nurse will explain the study objectives and procedures. The patient will review the informed consent form and sign it.

##### Baseline Questionnaire

Participants will fill out the questionnaires (Multimedia Appendix 1) to provide information on demographics, oral health, and previous history of surgeries and hospitalizations in the past year.

##### Blood Collection

A trained nurse will perform a standard venipuncture, drawing the required blood tubes for the chemistry parameters and cytokine analysis. Upon completion, participants will answer a short questionnaire assessing their experience and impression of the blood draw. This questionnaire will be repeated after each sampling method (Multimedia Appendix 2). The blood samples will be centrifuged and aliquoted into cryovial vials according to the laboratory instruction, frozen within 1 hour of collection, and preserved in sterile tubes at −80 °C until analysis.

##### Urine Collection

Midstream urine samples will be collected in sterile containers. Immediately after collection, we will perform a dipstick test (ie, Roche Combur Test Strip) to exclude samples with relevant bacterial contamination. The suitable samples will be centrifuged and aliquoted into cryovial vials according to the laboratory instruction, frozen within 1 hour of collection, and preserved in sterile tubes at −80 °C until analysis.

##### Core Body Temperature Sensor Placement

The CDHI researcher will attach a core body temperature sensor (eg, greenTEG CORE) to the upper body or chest area approximately 20 cm below the armpit using a heart rate monitor strap or medical patch. The sensor will be collected at the follow-up meeting.

##### Saliva Collection

Saliva samples are collected using an absorbent swab. Participants will place an absorbent swab (ie, Salimetrics SalivaBio Oral Swab) underneath the tongue for 3 minutes followed by direct disposal to a collection tube. Salivary samples will be transported to a storage unit within 1 hour of measurement and refrigerated at –80 °C until the analysis.

##### Sweat Patch Placement

The CDHI researcher will attach 2 sweat patches (ie, PharmChek Sweat Patch) on the participants’ arms or abdomen for sweat collection following the manufacturer’s instructions. Participants will be informed of the dos or do nots for the next 48 hours. The sweat patches will be collected at the follow-up meeting.

##### Exhaled Breath Test

Participants will complete FeNO measurements in exhaled breath using the “Bosch Vivatmo me” device. All measurements will be repeated twice per participant. “Bosch Vivatmo me” is a breathalyzer designed to measure airway inflammation based on the FeNO level detected in exhaled breath.

##### Stool Sampling Instruction

Participants will be provided a guide for collecting stool samples.

#### Visit 2 (Follow-Up Meeting)

Participants will be scheduled for a 20-minute follow-up meeting after 48 hours after study measurements. At this meeting, the CDHI researcher will remove the sweat patch and the core body temperature sensor, collect the stool sample, and conduct an interview on overall experience and preferences comparing sampling methods (Multimedia Appendix 3). Collected sweat patches and stool samples will be refrigerated at –80 °C until the analysis. The follow-up meeting will occur either in the patient’s room or the clinical examinations room available on the ward.

#### Patient Instructions

If participants agree to participate in this research project, they are asked to adhere to the specifications and requirements of the research project through the protocol. First, from 2 hours before study measurement to saliva collection: refrain from eating, drinking coffee, or acidic or sweet liquids, chewing gums, brushing teeth, using mouthwash and smoking. Second, from study measurements to follow-up meetings: avoid hot tubs, tanning booths, prolonged exposures in a sauna, or sweat lodges. Normal activities such as bathing, showering, swimming, and exercising are permitted. When drying the area where the sweat patch is applied, gently pat the area with a towel instead of rubbing the area.

### Measurements

#### End Points

**Figure 2:**
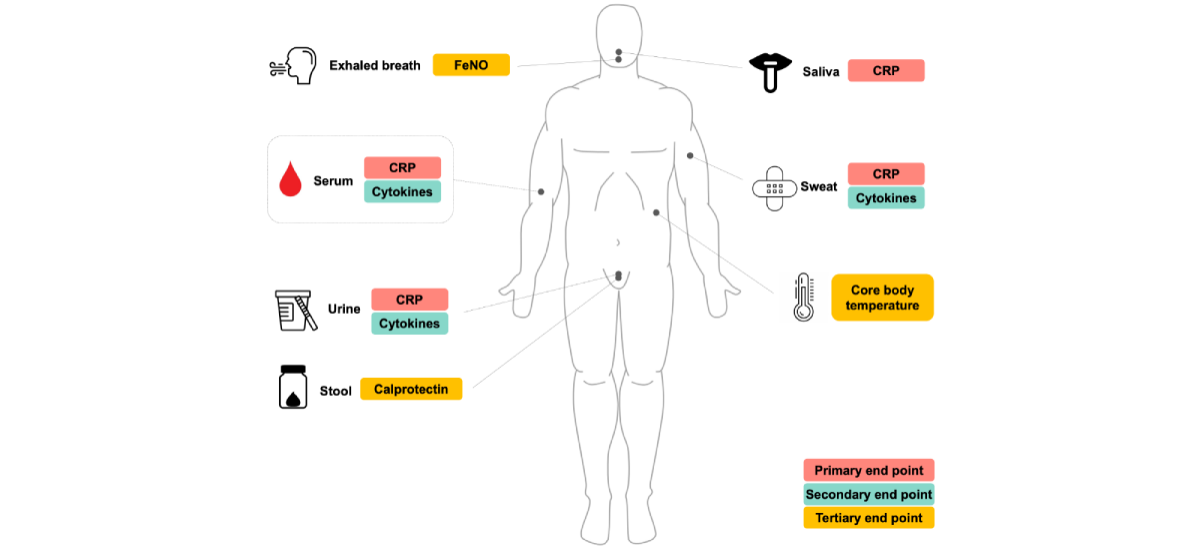
An overview of study measurements and end points. CRP: c-reactive protein; FeNO: fractional exhaled nitric oxide.

The primary endpoint of this study is the level of CRP. Secondary end points are the levels of inflammatory cytokines such as IL-1β, IL-6, IL-8, IL-10, and TNF-α. Tertiary end points are FeNO, calprotectin, and core body temperature (Figure 2).

#### Additional Information

As described in Textbox 1, we will collect additional information on patient characteristics and demographics, disease characteristics (eg, disease diagnosis, age at diagnosis, disease duration, and comorbidities), medications (eg, type, duration, and dose), measures of disease activity and functional outcomes (eg, Disease Activity Score-28 with CRP for rheumatoid arthritis, Bath Ankylosing Spondylitis Disease Activity Index for spondyloarthritis, Disease Activity in Psoriatic Arthritis for psoriatic arthritis, and Systemic Lupus Erythematosus Disease Activity for systemic lupus), oral health (eg, fasting, teeth status, and presence of oral diseases), and previous histories of surgeries and hospitalizations. To standardize the results of measured biomarkers, we will measure creatinine, albumin, and total protein levels in serum and urine and report the excretion of each urinary protein as a ratio between the concentration of the respective protein and urinary creatinine in a similar manner to albumin and creatinine ratio.

**Additional information.**

**Baseline characteristics**

- Demographics: age, sex, race, marital status, employment status, education, weight, height, BMI, blood pressure, smoking status, and comorbidities

**Disease characteristics**

- Disease diagnosis, age at diagnosis, disease duration, disease activity, and functional outcomes

**Medications**

- Type, duration, and dose

**Oral health**

- Status of fasting, brushing teeth, chewing gums, smoking, and drinking
- Bleeding gums and presence of oral diseases

**Prior history**

- Hospitalization within 1 year
- Surgical operation within 1 year

**Sample standardization**

- Creatinine, albumin, and total protein levels in serum
- Creatinine, albumin, and total protein levels in urine

### Sample Size Calculation

Since there is no previous knowledge or evidence of the outcome measures in this context, we calculate the sample size based on the following assumptions and considerations. First, we compute the sample size based on the serum CRP level to make sure that differences in CRP level are significant between both study groups. Second, we refer to studies of adult patients diagnosed with rheumatoid arthritis, which is one of the most prevalent systemic inflammatory diseases. CRP also plays a pivotal role in disease assessment and inflammatory activity in rheumatoid arthritis, which makes it a suitable choice of disease for exploring the sample size [14,15].

Bechman et al [16] reported that the serum CRP levels in patients with rheumatoid arthritis in systemic inflammatory states (ie, medium and high disease activity) range from 5 to 9 (SD 3.0) mg/L. Lillegraven et al [17] reported that the serum CRP levels in patients in the nonsystemic inflammatory state (ie, remission) range from 2 to 3 mg/L. Based on these results, it is plausible to conclude that we will observe a 3 to 6 mg/L difference in serum CRP levels comparing systemic inflammation versus nonsystemic inflammation among patients with rheumatoid arthritis. On this basis, and assuming 80% of power, 5% of type I error, and equal variance in groups [18], a sample size of 10 participants per group is required to detect a difference of 4 (SD 3.0) mg/L in serum CRP levels with (Table 1). Considering dropout and missing data, the sample size required may be increased by 10% to 20%.

**Table 1:** Sample size calculation.

Difference of means between groups	Pooled SD and sample size				
	SD 1.0	SD 2.0	SD 3.0	SD 4.0	SD 5.0
2 mg/L	6	17	37	64	100
3 mg/L	4	9	17	29	45
4 mg/L	3	6	10	17	26
5 mg/L	3	4	7	12	17

### Statistical Analysis

Descriptive analysis will report the mean and SD for continuous variables and the count and percentage for categorical variables. The 2-tailed *t* test and the chi-square test (or Fisher exact test if expected n<5) will be used for univariable analysis. In addition, the Shapiro-Wilk test will be used to evaluate the normality of the distribution in each variable. We will perform the 2-tailed *t* test (or Wilcoxon rank-sum test, if nonnormal) to compare the levels of inflammatory biomarkers between patients with systemic inflammations and controls. Subsequently, analyses of covariance or multiple regressions will be conducted to examine whether the mean biomarker level differs significantly between patients with systemic inflammation versus controls, adjusting for confounding factors such as baseline characteristics and oral health. Secondary and tertiary end points will be analyzed analogously. We will compute Pearson and Spearman correlation coefficients to assess the relationship between inflammatory biomarkers from noninvasive methods and serum biomarkers. For core body temperature data, the repeated measures correlation method will be performed to assess the association [18]. Participants’ preferences for 7 sampling methods will be assessed using a questionnaire, with rankings quantified on a scale from 1 (most preferred) to 7 (least preferred). To determine if combining noninvasive methods improves predictive accuracy of systemic inflammation, we will use an all-subset regression analysis. The combination of methods that yields the highest adjusted *R*^2^ values for predicting serum CRP levels will be considered the optimal model. Statistical analysis will be performed with R (R Core Team) or Python (Python Software Foundation). Statistical significance is set at 2-sided *P*<.05.

### Biomarker Analysis

**Figure 3:**
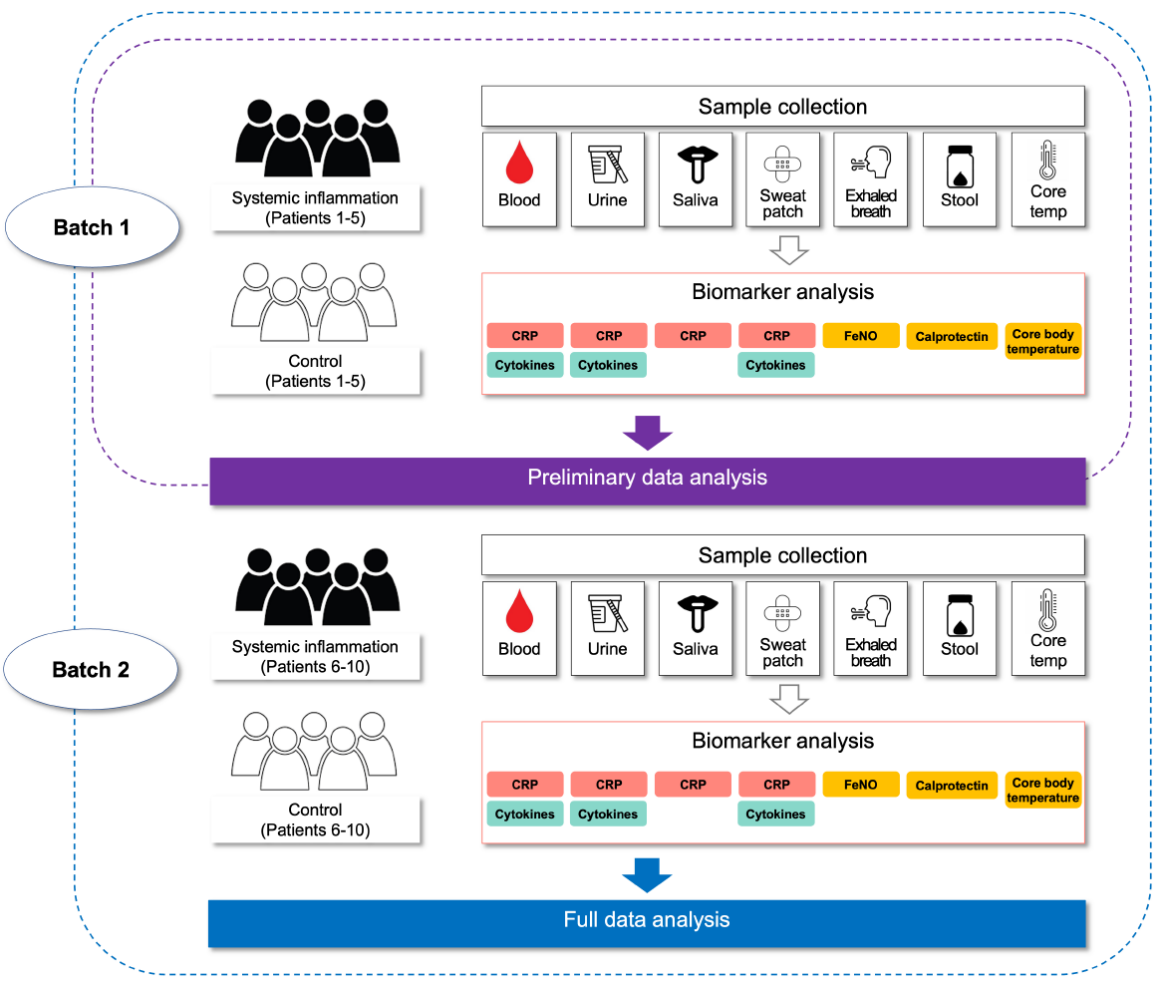
Proposed biomarker analysis. CRP: c-reactive protein; FeNO: fractional exhaled nitric oxide.

Due to the pilot nature of this study, it is difficult to estimate the precise time required to complete study recruitment and data collection. To overcome this limitation and enable preliminary data analysis, samples will be collected and submitted for biomarker analysis in 2 batches as described in Figure 3. Samples from the first half of the participants (ie, patients 1-5 from each group) will be collected and submitted to laboratories for the biomarker analyses (batch 1). Batch 2 will consist of samples from the second half of the participants (ie, patients 6-10 from each group). This will provide us with information on the accuracy of our estimated sample size and allow us to make corrections with regard to the planned number of patients to be included. Full data analysis will be conducted including the entire samples. Serum CRP, serum cytokines, and fecal calprotectin will be analyzed in the in-house laboratories of University Hospital Zurich according to the laboratory procedures. The levels of urinary CRP, urinary cytokines, salivary CRP, sweat CRP, and sweat cytokines will be determined at Swiss BioQuant. Noninvasive CRP levels will be quantified by the enzyme-linked immunosorbent assay according to instructions provided by the manufacturer. Noninvasive cytokines will be analyzed using electrochemiluminescence immunoassay.

### Data Management

Project data will be handled with the uttermost discretion and are only accessible to authorized personnel who require the data to fulfill their duties within the scope of the research project. Participant data will be encrypted. The participant identification list will be stored separately from the study records and only accessible by a dedicated data manager, who was not involved in the study. On the case report forms and other project-specific documents, participants are only identified by a unique participant number. Access and changes to documents and data servers will be logged and traced. Data will further be backed up on encrypted drives and safeguarded in a key-locked cupboard.

### Ethical Consideration

This study received ethical approval from the independent research ethics committee of Canton Zurich on October 28, 2022 (BASEC-Nr: 2022-01386). This research project will be conducted in accordance with the protocol, the Declaration of Helsinki, the principles of good clinical practice, the Human Research Act, and the Human Research Ordinance, as well as other locally relevant regulations. All participants will provide written informed consent before they participate in the study. Participation in the study is fully voluntary, and withdrawal from the study is possible at any time.

## Results

The study received ethics approval from the independent research ethics committee of Canton Zurich on October 28, 2022. A total of 20 participants participated in the study measurements. Data collection started on February 22, 2023, and was completed on September 22, 2023. Participants were on average 52.8 (SD 14.4, range 24-82) years of age, and 70% (14/20) of them were women. The analysis results reporting findings are expected to be published in 2025.

## Discussion

### Principal Findings

This study aims to investigate the noninvasive assessment of inflammatory biomarkers in patients with systemic inflammation. We hypothesize that inflammatory biomarkers measured noninvasively in urine, sweat, saliva, stool, core body temperature, and exhaled breath are significantly associated with serum biomarker levels. The study will also examine if the multimodal, noninvasive approach yields stronger associations and increases the predictive ability of serum biomarker levels compared with a single noninvasive method. Furthermore, we anticipate that assessing individual preferences for different noninvasive methods provides valuable insights into patients’ acceptance when implemented in real-life settings.

### Comparison to Previous Work

Prior work has explored the detection of inflammatory biomarkers in urine, saliva, and sweat. Researchers reported that elevated urinary TNF-α level is correlated with chronic inflammation and endothelial dysfunction in obese adolescents [19]. A study by Galhardo et al [20] measured CRP and IL-6 in saliva to predict sepsis diagnosis in hospitalized patients in an intensive care unit setting. Hladek et al [21] analyzed inflammatory cytokines including IL-6, IL-10, and TNF-α collected using sweat patches among 49 young and older adults. More recently, researchers developed a wireless patch for the real-time electrochemical detection of the CRP in sweat and assessed the evaluation in patients with chronic obstructive pulmonary disease [22]. While these studies have provided valuable insights, they implement a single biofluid modality, where detection accuracy may be highly variable depending on the presence of local inflammation (eg, urinary tract infection), environmental factors (eg, ambient temperature), or biomarker characteristics (eg, diurnal variation). Furthermore, previous research has primarily involved healthy individuals or specific disease types, with limited work addressing systemic inflammation. Our study differs by adopting a multimodal approach using easy-to-collect samples and focusing specifically on patients with systemic inflammation, thus addressing a significant gap in the existing literature.

### Study Implications

Systemic inflammation is a major contributor to chronic inflammatory diseases, which are responsible for more than 50% of worldwide deaths today. Given its low-grade, persistent, and chronic nature, continuous monitoring and early diagnosis of exacerbations are especially important for patients with systemic inflammation. Currently, there is no scalable solution for remote assessment of inflammatory status, often leading many patients to delay timely interventions until symptoms become severe or until their next routine visit. Although clinical guidelines for disease management and treatment are well established, many patients still encounter unanticipated exacerbations resulting in pain, worsened symptoms, and reduced quality of life. Thus, this proof-of-concept study provides an important avenue for exploring the feasibility of noninvasive inflammation assessment. To our knowledge, this is the first study to simultaneously collect and measure inflammatory biomarkers from 6 different noninvasive methods and evaluate their associations with serum biomarkers. Establishing these relationships would provide the basis for digitizing measurement for serum biomarkers, such as for CRP, contributing to the creation of a digital surrogate biomarker. Once digitized, this approach could lead to the development of a scalable, cost-efficient sensor, allowing a timely and rapid monitoring of inflammatory activity. This advancement is particularly relevant given the ever-rising incidence of systemic inflammatory conditions and the urgent need for precise and remote monitoring tools.

### Strengths and Limitations

A key strength of our study is that we collect 6 distinct types of body fluids in addition to blood from the same individual and perform a biomarker immunoassay assessment through comprehensive testing. A proposed multimodal approach not only increases the precision and sensitivity of detected associations but also offers new insights into patients’ experiences or preferences related to different sampling methods. Second, we include different types of inflammatory biomarkers (ie, CRP, interleukins, calprotectin, FeNO, and core body temperature) as the sensitivity of detection varies by molecules and biofluid types. As opposed to existing research with a single outcome, we seek to contribute to the current literature by assessing multiple endpoints that have been acknowledged to have a direct or indirect association with the gold standard. Third, we minimize an unanticipated temporal or circadian influence or medication effect in the inflammatory state by obtaining blood and noninvasive samples simultaneously. To achieve simultaneous sampling, we propose an efficient workflow of study procedure that enables us to complete study measurements and the follow-up meeting within a total of 1 hour. Fourth, it is the first to implement noninvasive, continuous monitoring of core body temperature for 48 hours to associate the temporal pattern of body temperature with inflammatory activities in systemic inflammation. Finally, we obtain additional information on patient and disease characteristics, disease activity, medications, comorbidities, and oral health questionnaires and adjust statistical models for potential confounding.

This study will have limitations. With the pilot nature of the study, no longitudinal measurements will be collected. Given the nature of the cross-sectional study, we are not able to establish causal relationships from observed associations. In addition, our study involves a relatively small size at a single study site among hospitalized patients at a University hospital, which may limit the generalizability of our findings to a real-world patient population. Nevertheless, this proof-of-concept study offers novel insights into the digitization of inflammatory biomarkers and directions for future research opportunities. If this proof-of-concept study yields successful results, a longitudinal, prospective cohort study is warranted.

### Dissemination Plan

We are committed to reporting the study results through peer-reviewed journals and presentations to disseminate key findings and learnings to various clinical and scientific communities.

### Conclusion

This study aims to evaluate the feasibility of noninvasive, multimodal assessment of inflammatory biomarkers in patients with systemic inflammation. Promising results could lead to the creation of noninvasive and potentially digital biomarkers, enabling continuous monitoring and early diagnosis of inflammatory activity in a remote setting.

## Appendix

Multimedia Appendix 1 Baseline questionnaire (in German).

Multimedia Appendix 2 Questionnaire on assessment of sampling methods (in German).

Multimedia Appendix 3 Questionnaire on preference for sampling methods (in German).

## Abbreviations

CDHI: Centre for Digital Health Intervention
CRP: C-reactive protein
FeNO: fractional exhaled nitric oxide
IL: interleukin
TNF: tumor necrosis factor

## Data Availability

The datasets generated and analyzed during this study are not publicly available due to patient confidentiality but may be available from the corresponding author upon reasonable request.
