# Supplementary material for: Noninvasive, Multimodal Inflammatory Biomarker Discovery for Systemic Inflammation (NOVA Study): Protocol for a Cross-Sectional Study
Source: JMIR Res Protoc. 2024 Nov 5;13:e62877. doi: 10.2196/62877 (PMC11576606; doi:10.2196/62877)
Supplement: Multimedia Appendix 1 [file resprot_v13i1e62877_app1.pdf]

Multimedia Appendix 1. Baseline Questionnaire (in German)

Demografische Daten

1. Wie alt sind Sie?

   __________ Jahre alt

2. Welches ist Ihr Geschlecht?
   - ☐ Männlich
   - ☐ Weiblich

3. Welcher Rasse gehören Sie an?
   - ☐ Afroamerikanisch
   - ☐ Kaukasisch
   - ☐ Asiatisch
   - ☐ Andere: ____________________

4. Was ist Ihr Zivilstand?
   - ☐ Ledig (nie verheiratet)
   - ☐ Getrennt lebend
   - ☐ Verheiratet
   - ☐ Verwitwet
   - ☐ Geschieden
   - ☐ Andere: ____________________

5. Wie ist Ihr Arbeitsverhältnis?
   - ☐ Vollzeit
   - ☐ Teilzeit
   - ☐ Im Ruhestand
   - ☐ Selbstständig
   - ☐ Nicht erwerbstätig
   - ☐ Andere: ____________________

6. Welchen höchsten Bildungsabschluss haben Sie erreicht?
   - ☐ unvollständige Sekundarschule/Oberstufe
   - ☐ Master-Abschluss
   - ☐ Sekundarschule/Oberstufe
   - ☐ Ph.D. oder höher
   - ☐ Bachelor-Abschluss
   - ☐ Andere: ____________________

Mundgesundheit

1. Sind Sie nüchtern? (Kein Essen oder Trinken ausser Wasser in den letzten 2 Stunden)
   - ☐ Ja
   - ☐ Nein

   Falls nein, listen Sie auf, was Sie gegessen oder getrunken haben: ____________________

2. Haben Sie sich in den letzten 2 Stunden die Zähne geputzt oder eine Mundspülung benutzt?
   - ☐ Ja
   - ☐ Nein

3. Haben Sie in den letzten 2 Stunden Kaugummi gekaut?
   - ☐ Ja
   - ☐ Nein

4. Haben Sie in den letzten 2 Stunden geraucht?
   - ☐ Ja
   - ☐ Nein

5. Haben Sie in den letzten 24 Stunden Alkohol getrunken?
   - ☐ Ja
   - ☐ Nein

6. Haben Sie in den letzten 24 Stunden jemals Zahnfleischbluten bemerkt, wenn Sie nicht geputzt haben?
   - ☐ Ja
   - ☐ Nein

7. Leiden Sie derzeit an einer der folgenden Mundkrankheiten? Parodontitis und Gingivitis, Parodontitis?

☐ Ja ☐ Nein

Wenn ja, sind Sie derzeit deswegen in Behandlung?

☐ Ja ☐ Nein

Frühere Krankenhausaufenthalte und Operationen

1. Wurden Sie im letzten Jahr wegen eines entzündlichen Krankheitsschubs ins Krankenhaus eingeliefert?

☐ Ja ☐ Nein

2. Wenn "Ja", wie hoch ist die Gesamtzahl der Krankenhausaufenthalte im letzten Jahr?

________________________ TAGE

3. Hatten Sie im letzten Jahr irgendwelche Operationen?

☐ Ja ☐ Nein

4. Wenn "Ja", wie viele Operationen waren es insgesamt im letzten Jahr?

____
