# Supplementary material for: Noninvasive, Multimodal Inflammatory Biomarker Discovery for Systemic Inflammation (NOVA Study): Protocol for a Cross-Sectional Study
Source: JMIR Res Protoc. 2024 Nov 5;13:e62877. doi: 10.2196/62877 (PMC11576606; doi:10.2196/62877)
Supplement: Multimedia Appendix 2 [file resprot_v13i1e62877_app2.pdf]

Multimedia Appendix 2. Questionnaire on Assessment of Sampling Methods (in German)

Anweisung an die Patienten: Bitte geben Sie Ihre Zustimmung zu den Attributen an, indem Sie den Kreis ankreuzen, der Ihrem Eindruck von der soeben durchgeführten Messmethode am ehesten entspricht.

**Blut**	**Q1**	Angenehm	○ ○ ○ ○ ○	Lästig
	**Q2**	Einfach	○ ○ ○ ○ ○	Kompliziert
	**Q3**	Effizient	○ ○ ○ ○ ○	Ineffizient
	**Q4**	Weniger belastend	○ ○ ○ ○ ○	Belastend
	**Q5**	Leicht zu lernen	○ ○ ○ ○ ○	Schwierig zu lernen
**Urin**	**Q1**	Angenehm	○ ○ ○ ○ ○	Lästig
	**Q2**	Einfach	○ ○ ○ ○ ○	Kompliziert
	**Q3**	Effizient	○ ○ ○ ○ ○	Ineffizient
	**Q4**	Weniger belastend	○ ○ ○ ○ ○	Belastend
	**Q5**	Leicht zu lernen	○ ○ ○ ○ ○	Schwierig zu lernen
**Schweiss-pflaster**	**Q1**	Angenehm	○ ○ ○ ○ ○	Lästig
	**Q2**	Einfach	○ ○ ○ ○ ○	Kompliziert
	**Q3**	Effizient	○ ○ ○ ○ ○	Ineffizient
	**Q4**	Weniger belastend	○ ○ ○ ○ ○	Belastend
	**Q5**	Leicht zu lernen	○ ○ ○ ○ ○	Schwierig zu lernen
**Speichel**	**Q1**	Angenehm	○ ○ ○ ○ ○	Lästig
	**Q2**	Einfach	○ ○ ○ ○ ○	Kompliziert
	**Q3**	Effizient	○ ○ ○ ○ ○	Ineffizient
	**Q4**	Weniger belastend	○ ○ ○ ○ ○	Belastend
	**Q5**	Leicht zu lernen	○ ○ ○ ○ ○	Schwierig zu lernen
**Atemluft-unter-suchung**	**Q1**	Angenehm	○ ○ ○ ○ ○	Lästig
	**Q2**	Einfach	○ ○ ○ ○ ○	Kompliziert
	**Q3**	Effizient	○ ○ ○ ○ ○	Ineffizient
	**Q4**	Weniger belastend	○ ○ ○ ○ ○	Belastend
	**Q5**	Leicht zu lernen	○ ○ ○ ○ ○	Schwierig zu lernen
	**Q1**	Angenehm	○ ○ ○ ○ ○	Lästig

**Sthul**	**Q2**	Einfach	◯ ◯ ◯ ◯ ◯	Kompliziert
	**Q3**	Effizient	◯ ◯ ◯ ◯ ◯	Ineffizient
	**Q4**	Weniger belastend	◯ ◯ ◯ ◯ ◯	Belastend
	**Q5**	Leicht zu lernen	◯ ◯ ◯ ◯ ◯	Schwierig zu lernen
**Körperkerntemperatur**	**Q1**	Angenehm	◯ ◯ ◯ ◯ ◯	Lästig
	**Q2**	Einfach	◯ ◯ ◯ ◯ ◯	Kompliziert
	**Q3**	Effizient	◯ ◯ ◯ ◯ ◯	Ineffizient
	**Q4**	Weniger belastend	◯ ◯ ◯ ◯ ◯	Belastend
	**Q5**	Leicht zu lernen	◯ ◯ ◯ ◯ ◯	Schwierig zu lernen
