# Supplementary material for: Noninvasive, Multimodal Inflammatory Biomarker Discovery for Systemic Inflammation (NOVA Study): Protocol for a Cross-Sectional Study
Source: JMIR Res Protoc. 2024 Nov 5;13:e62877. doi: 10.2196/62877 (PMC11576606; doi:10.2196/62877)
Supplement: Multimedia Appendix 3 [file resprot_v13i1e62877_app3.pdf]

Multimedia Appendix 3. Questionnaire on Preference for Sampling Methods (in German)

Q1. Basierend auf Ihren Erfahrungen in dieser Studie, ordnen Sie bitte jede der folgenden Messmethoden in der Reihenfolge ihrer Präferenz ein (1 - am meisten bevorzugt, 7 - am wenigsten bevorzugt). Bitte lassen Sie das Kästchen leer, wenn Sie nicht an den Messungen teilgenommen haben.

Blut	Urin	Schweiss-pflaster	Speichel	Atemluft-unter-suchung	Stuhl	Körperkerntemperatur
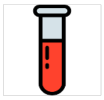	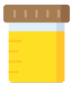	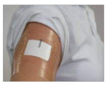	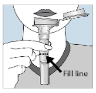	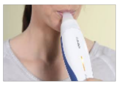	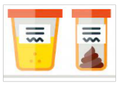	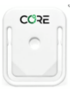

Q2. Warum haben Sie diese Reihenfolge gewählt? Bitte erklären Sie das.

Q3. Können Sie mir nun sagen, welche Erfahrungen Sie insgesamt mit der Teilnahme an dieser Studie gemacht haben? Gute oder schlechte Dinge, das Beste oder das Schlimmste, was gut und was nicht gut funktioniert hat.

Q4. Warum haben Sie sich für die Teilnahme an dieser Studie entschieden?

Q5. Haben Sie noch weitere Anmerkungen zu machen?
